# Announcement: The Third International Symposium on Applied Bioinorganic Chemistry

**DOI:** 10.1155/MBD.1994.291

**Published:** 1994


					THE THIRD
INTERNATIONAL SYMPOSIUM
ON APPLIED BIOINORGANIC
CHEMISTRY
Bioinorganic chemistry has increasing impact in many areas of science and
technology. Following the successful first two Symposia in China (Wuhan, 1898;
Guangzhou, 1992), the Third INTERNATIONAL SYMPOSIUM ON
APPLIED BIOINORGANIC CHEMISTRY (ISABC) will focus on these applied
aspects of bioinorganic chemistry. Themes include metal-containing frugs,
radiopharmaceuticals, toxicology and nutrition, biologically active chelates and
macrocycles, biominerals and new materials, as well as environmental, marine
and agricultural aspects. The Proceedings will be published internationally to
disseminate this perspective to the wider scientific community.
Conference chairs"
Professor Wang Kui (Beijing Medical University)
and
Associate Professor John Webb (Murdoch University)
Dates: December 11 16, 1994
(prior to the 4th EURASIA Conference, Kuala Lumpur, Malaysia, December 17 22)
Location: PERTH, WESTERN AUSTRALIA
The Conference Venue is Fremantle, within the Perth metropolitan area, and
which is a Victorian-era seaport at the mouth of the Swan River. It is renowned for
its marine streetside coffee houses, cosmopolitan restaurants, theatres, arts and
craft markets, shopping facilities and tourist attractions.
For information and offer of papers
CONTACT: Associate Professor John WEBB,
School of Mathematical and Physical Sciences,
Murdoch University, Murdoch, Western Australia 6150.
Telephone: 61 9 360 2148
Facsimile" 61 9 310 ,5005
e-mail" johnwebb@vaxl .csu.murdoch.edu.au
291

				

